# “Healthy Young People Mean Healthy Societies”: Focus on Mental Health and Mental Illness in Adolescents

**DOI:** 10.3390/brainsci16030253

**Published:** 2026-02-24

**Authors:** Dariusz Krok

**Affiliations:** Institute of Psychology, University of Opole, 45-040 Opole, Poland; dkrok@uni.opole.pl

## 1. Introduction

In recent years, we have observed a clear and dramatic increase in mental health difficulties and psychological problems among young people [1,2]. This trend is associated with numerous psychological, social, cultural, and technological factors. As a consequence, young people are exposed to rapid and dynamic changes in their mental health. Every illness—particularly mental illness—has a profound impact on an individual’s psychosocial functioning and requires considerable strength and determination to overcome the limitations and challenges of everyday life. Mental illness affects the individual as a whole, across physical, psychological, and social domains, leading to a range of profound changes in thinking, emotional experience, and behavior [3]. The negative effects of mental illness are especially noticeable in young people, due to ongoing developmental changes, the intensity of emotional experiences that are characteristic of this life stage, high levels of stress, and social challenges. As a result, there is a growing demand for the development of psychological research and interventions directed at young people, as well as an increasing need for research within health psychology that is focused on mental health and mental disorders in adolescents.

Adolescence represents a particularly vulnerable developmental period, characterized by rapid biological maturation, identity formation, emotional reactivity, and increased sensitivity to social evaluation, all of which may heighten susceptibility to mental health difficulties [4]. Epidemiological data indicate a rising prevalence of anxiety disorders, depression, behavioral problems, and stress-related symptoms among adolescents, underscoring the urgency of addressing mental health during this formative stage of life [5,6]. Mental health and mental illness in adolescents have emerged as critical areas of research and major social concerns in recent decades, driven by a complex interplay of social, technological, biological, and environmental factors. Research shows that one of the main causes responsible for the sudden decline in mental health among adolescents was the COVID-19 pandemic, which triggered a number of detrimental effects related to social relations (e.g., loneliness, isolation, lack of social interactions) [7,8,9]. This is because adolescents are among the populations that are most vulnerable to the preventive measures implemented during the COVID-19 pandemic, including school closures, limited peer interaction, and reduced opportunities for physical activity and exploration. These restrictions tended to have a series of negative effects upon adolescents’ well-being and mental health, contributing to an increased risk of sleep disturbances, depressive symptoms, heightened stress, and anxiety.

Mental illnesses in adolescents arise from multifactorial causes, encompassing genetic, biological, psychological, social, and familial influences. They are involved in multiple, complex interactions that shape developmental patterns and underlying mechanisms. Genetic vulnerability may include a family history of mental illness or specific genetic variations that increase sensitivity to stress and emotional dysregulation [10]. Recent research revealed that biological factors also take part in this negative process, including neurodevelopmental processes, hormonal changes associated with puberty, the impact of chronic physical illness, sleep disturbances, and the misuse of psychoactive substances, all of which can significantly affect psychological functioning [11]. The development of mental illness is rooted in abnormalities in genetic information, as a result of which the brain develops and functions in an uncontrolled and distorted manner, and the existing brain structures fail to perform the roles that are appropriate for their tissue of origin. Consequently, maladaptive neural structures emerge in the brain that threaten its proper functioning, and they elicit responses across physiological, psychological, and social levels; as a result of this, the physiological development of a young person becomes disrupted.

This indicates that genetic and biological determinants constitute a significant component of mental illness etiology among adolescents. The presence of inherited genes that increase the risk of developing serious mental issues to a lesser or greater extent represents an unfavorable factor that cannot be eliminated. However, an important conclusion drawn from the above-mentioned research findings is the possibility of an interaction between, on one hand, genetic and biological factors, and on the other hand, psychological and environmental factors in the development of mental disorders. As a consequence, there is a clear need to consider a broad range of factors beyond genes and biological mechanisms alone.

In addition to biological and genetic determinants, psychosocial and environmental factors play a central role in adolescent mental health [12,13]. Psychological and environmental influences may either attenuate existing genetic predispositions (e.g., a balanced diet and a healthy lifestyle) or exacerbate them (e.g., exposure to stress or lack of social support) [14]. Research has consistently pointed to several detrimental factors in adolescents’ mental health. Lifestyle changes, including reduced physical activity, irregular sleep patterns, and excessive screen time, are often linked to poorer emotional well-being. The pervasive influence of social media and digital technologies introduces new stressors, such as cyberbullying, social comparison, fear of missing out, and constant exposure to idealized images, which may negatively affect self-esteem and emotional regulation. Academic pressures, including high performance expectations, competitive educational environments, and concerns about future employment, further contribute to elevated stress levels among adolescents [15,16,17]. All these factors are very likely to generate severe stress that may exceed the adaptive capacities of a young person and hinder normal development. This substantially impairs the psychological functioning of young people and diminishes their mental health.

Moreover, many adolescents are exposed to adverse experiences within their families and communities, such as domestic violence, parental substance abuse, neglect, or community-level violence. These family experiences can undermine their emotional security and increase their risk of internalizing and externalizing problems [18]. As a consequence, young people may become detached from normal developmental processes and experience a number of serious mental issues. Finally, rapid sociocultural changes—such as shifting family structures, evolving social norms, and global uncertainty—create additional challenges for adolescents as they navigate developmental tasks and construct a stable sense of identity. Taken together, these factors highlight the need for comprehensive, developmentally informed research and intervention strategies to promote mental health and resilience among young people.

## 2. The Content-Related Scope of Published Articles

This Special Issue aims to explore and identify the determinants, consequences, and mechanisms associated with mental health and mental illness among adolescents. Consistently with the previously cited research findings, mental health issues can profoundly impact an adolescent’s development. Therefore, examining mental health and mental illness in adolescents is crucial to ensure their well-being, academic success, and healthy development into adulthood. The following articles clearly addressed these challenges and supplemented the previous findings.

The article published by Timoszyk-Tomczak et al. (Contribution 1) examined feelings of loneliness—including intimacy, social connections, and belonging—among adolescents and their potential associations with some aspects of family relationships (e.g., communication, cohesion, autonomy–control, and identity). Based on methods assessing loneliness, anxiety, and family interactions, the results showed that the anxiety and adverse family relationship factors were important predictors of adolescent loneliness. In addition, communication, cohesion, autonomy–control, and identity within the family context significantly accounted for the experience of loneliness. This indicates that distinct dimensions of family interactions contribute to adolescents’ experiences of loneliness, underscoring the multilayered nature of their social lives.

Kroplewski et al.’s article (Contribution 2) focused on anxiety disorders and social support among young people aged 18–28 with a non-generalized form of social anxiety. This is important, as anxiety disorders are one of the most common mental disorders in young individuals and are often associated with significant discomfort and impaired functioning. The aim of this experimentally designed study was to investigate the impact of role-playing games (RPGs) as a support strategy for individuals with social anxiety disorder. The findings revealed a statistically significant reduction in anxiety and avoidance in all groups, with the greatest changes being observed in the total support score. This implies that role-playing game interventions may help young people with social anxiety to receive support, leading to symptom reduction and better social functioning.

The next article (Szcześniak et al.; Contribution 3) investigated mediational mechanisms underlying the associations between self-efficacy, loneliness, and peer communication. A total of 191 primary and high school students completed three scales assessing general self-efficacy, loneliness, and communication patterns with peers. Correlational analyses showed that self-efficacy was negatively associated with loneliness and difficulty in adolescents’ peer communication, but was positively associated with openness in adolescents’ peer communication. Furthermore, openness in adolescents’ communication with peers and communication difficulty mediated the relationship described above. These findings point to a notable conclusion: namely, young people who strongly believe in their ability to succeed are more likely to share personal experiences with others and to experience less difficulty in peer-group communication, which is associated with lower levels of loneliness.

Chmiel and Kroplewski’s study (Contribution 4) aimed to explore the associations between growth-oriented values (openness to change and self-transcendence) and psychological well-being among emerging adults, with meaning in life serving as a mediator. All the variables used in the study were positively correlated. The main finding demonstrated that the presence of meaning mediated the relationship between both openness to change and self-transcendence and psychological well-being. In contrast, the search for meaning was not a mediator in either model. This underscores the importance of growth-oriented values and the presence of meaning in the psychological dimensions of well-being among emerging adults.

The fifth article (Krok and Półtorak, Contribution 5) aimed to assess the mediating roles of Facebook addiction and perceived stress in the association between attitudes toward social media and psychological well-being among emerging adults. After conducting correlation and mediation analyses, most hypotheses were confirmed. Facebook addiction and perceived stress were found to serially mediate between attitudes toward social media and well-being, which indicates that the enhancement of agency in young adults may serve as a protective factor against the development of Facebook addiction, and thus may alleviate stress and ultimately promote better psychological well-being and overall mental functioning.

The sixth study by Yousef et al. (Contribution 6), published in this Special Issue, investigated the phenomenon of “brain rot” in the digital era through a thorough review of prior studies. Given the widespread and increasing use of digital technologies, it is important to scrutinize multiple approaches, such as mindful technology use, to support cognitive functioning and emotional well-being. The review found that brain rot is inadvertently related to emotional desensitization, cognitive overload, and a negative self-concept, as well as several detrimental psychological consequences, including psychological distress, anxiety, and depression. There is therefore a need to address the ubiquitous impact of brain rot by supporting a balanced approach to technology use among adolescents and young adults.

In the seventh article, Chęć et al. (Contribution 7) examined the dimensions of perfectionism in adolescence in the context of mental health and CBT-based psychoeducation. After employing two complementary approaches—cross-sectional and experimental—the authors found that maladaptive perfectionism was positively associated with depression, anxiety, stress, and difficulties in emotional regulation, whereas adaptive perfectionism was negatively related to deficits in emotional understanding. In addition, psychoeducational interventions lowered maladaptive perfectionism and depression levels, but amplified stress.

The next study by Giordano et al. (Contribution 8) examined the potential role of maladaptive personality traits and post-traumatic stress symptomatology in adolescents’ perceptions of loneliness. The findings highlighted the significant role of post-traumatic stress disorder and disturbances in self-organization in experiences of loneliness, both directly and indirectly, through maladaptive personality traits. These results lead to the conclusion that adolescence is a crucial developmental period associated with a heightened risk of loneliness, as adolescents are expected to develop greater independence and more complex social relationships beyond their family context.

Examining scouting as a moderating strategy for supporting mental health, Szałachowski et al. (Contribution 9) explored the relationships between self-esteem, efficacy, and coping styles. It was found that scouting moderated the relationship between emotion-focused coping and self-efficacy, indicating that self-esteem blocks the relationship between emotion-focused coping and emotions during stressful situations experienced by young people. The study also highlights the significant role of scouting activities in fostering mature, satisfactory social relationships among adolescents who develop a pro-social orientation that is grounded in scouting values and ethical standards.

The next study by Chęć et al. (Contribution 10) delved into the topics of temperament, tension, and self-injurious behavior in adolescents from a mediational perspective. Perfectionism, sensory sensitivity, and emotional reactivity were found to increase the risk of self-injurious behavior. Furthermore, maladaptive perfectionism mediated the relationship between these traits and the propensity to experience emotional tension. This suggests the existence of a temperament profile that exerts a protective effect against the development of psychopathology that is linked to maladaptive perfectionism, intense emotional tension, and self-injurious behavior, as well as a distinct profile that predisposes individuals to self-injurious behavior and amplifies perceived emotional tension.

The last article in this Special Issue, written by Weiss et al. (Contribution 10) concerned the development of a therapeutic assessment protocol for the screening and support of young people’s mental health. The final outcome showed that the screening and feedback optimally assessed youth awareness in terms of well-being and/or distress, as well as their influence on daily functioning and well-being-oriented engagement. Implementing a multi-level suicide screening strategy, beginning at the community level, and following this up with individual assessments of suicide severity and risk identification, was found to be an efficient and effective method of suicide screening that also mitigated potential risks.

In total, eleven papers were finally accepted for publication and inclusion in this Special Issue. The contributions are listed below and their overview is presented in Table 1:

1. Timoszyk-Tomczak, C.; Pieńkowska, E.; Ligocka, M.; Piłat, M. Adolescents’ Feelings of Loneliness Considering Anxiety and Intrafamilial Relations. *Brain Sci.* **2025**, *15*, 1270. https://doi.org/10.3390/brainsci15121270.
2. Kroplewski, Z.; Łoś, R.; Pawlicki, B.J. Impact of Participation in Role-Playing Game (RPG) Sessions on the Perceived Level of Social Anxiety and Received Social Support. *Brain Sci.* **2025**, *15*, 1158. https://doi.org/10.3390/brainsci15111158.
3. Szcześniak, M.; Świątek, A.H.; Szczerba, A.; Szpunar, K.; Falewicz, A. General Sense of Perceived Self-Efficacy and Loneliness Among Polish Adolescents: Communication with Peers as Mediator. *Brain Sci.* **2025**, *15*, 946. https://doi.org/10.3390/brainsci15090946.
4. Chmiel, M.; Kroplewski, Z. Personal Values and Psychological Well-Being Among Emerging Adults: The Mediating Role of Meaning in Life. *Brain Sci.* **2025**, *15*, 930. https://doi.org/10.3390/brainsci15090930.
5. Krok, D.; Półtorak, M. Social Media Mindsets and Well-Being in Emerging Adults: A Serial Mediation of Facebook Addiction and Stress. *Brain Sci.* **2025**, *15*, 301. https://doi.org/10.3390/brainsci15030301.
6. Yousef, A.M.F.; Alshamy, A.; Tlili, A.; Metwally, A.H.S. Demystifying the New Dilemma of Brain Rot in the Digital Era: A Review. *Brain Sci.* **2025**, *15*, 283. https://doi.org/10.3390/brainsci15030283.
7. Chęć, M.; Konieczny, K.; Michałowska, S.; Rachubińska, K. Exploring the Dimensions of Perfectionism in Adolescence: A Multi-Method Study on Mental Health and CBT-Based Psychoeducation. *Brain Sci.* **2025**, *15*, 91. https://doi.org/10.3390/brainsci15010091.
8. Giordano, F.; Calaresi, D.; Saladino, V.; Verrastro, V. Perception of Loneliness in Adolescence: Role of Maladaptive Personality Traits and Trauma-Related Symptomatology. *Brain Sci.* **2025**, *15*, 86. https://doi.org/10.3390/brainsci15010086.
9. Szałachowski, R.R.; Własak, W.; Tuszyńska-Bogucka, W. Scouting as a Strategy in Support of Mental Health Development Through the Formation of Sense of Self-Efficacy. *Brain Sci.* **2024**, *14*, 1268. https://doi.org/10.3390/brainsci14121268.
10. Chęć, M.; Michałowska, S.; Gnych-Pietrzak, A.; Rybarska, A.; Strochalska, K. Temperament and the Experience of Tension and Self-Injurious Behaviour in Adolescents—The Mediating Role of Maladaptive Perfectionism. *Brain Sci.* **2024**, *14*, 1140. https://doi.org/10.3390/brainsci14111140.
11. Weiss, M.D.; Richards, E.C.; Bien-Aime, D.; Witkowski, T.; Williams, P.; Holmes, K.E.; Cortes, D.E.; Tepper, M.C.; Wang, P.S.; Aldis, R.; Carson, N.; Le Cook, B. The Development of a Brief but Comprehensive Therapeutic Assessment Protocol for the Screening and Support of Youth in the Community to Address the Youth Mental Health Crisis. *Brain Sci.* **2024**, *14*, 1134. https://doi.org/10.3390/brainsci14111134.

**Table 1:** Overview and the articles included in the Special Issue.

N# of Contribution	Research Area	Focus	Type of Research	Main Findings
1	Adolescent mental health; loneliness	Associations between loneliness, anxiety, and intrafamilial relations in adolescents	Empirical, cross-sectional study	Adolescent loneliness was strongly associated with anxiety and the quality of family relationships, indicating the protective role of supportive intrafamilial relations.
2	Social anxiety; social support	Effects of participation in role-playing game (RPG) sessions on social anxiety and perceived social support	Empirical, intervention-based study	Participation in RPG sessions was linked to reduced social anxiety and increased perceived social support, suggesting the therapeutic and social benefits of structured role-playing activities.
3	Self-efficacy; loneliness	Mediating role of peer communication in the relationship between self-efficacy and loneliness	Empirical study with mediation analysis	Peer communication mediated the association between perceived self-efficacy and loneliness, highlighting social interaction as a key mechanism linking personal resources to mental health outcomes.
4	Psychological well-being; values	Relationships between personal values, meaning in life, and psychological well-being in emerging adults	Empirical mediation study	Meaning in life mediated the relationship between personal values and psychological well-being, emphasizing the importance of existential factors for mental health.
5	Digital media; well-being	Impact of social media mindsets on well-being via Facebook addiction and stress	Empirical study with serial mediation	Maladaptive social media mindsets contributed to lower well-being indirectly, through increased Facebook addiction and stress, identifying digital behavior as a significant risk pathway.
6	Cognitive health; digital behavior	Conceptual analysis of “brain rot” in the digital era	Narrative review	Excessive digital engagement may negatively affect cognitive functioning and mental well-being, underscoring the need for mindful and balanced technology use.
7	Perfectionism; psychoeducation	Dimensions of perfectionism and their associations with mental health; CBT-based psychoeducation	Multi-method empirical study	Maladaptive perfectionism was associated with poorer mental health outcomes, while psychoeducation showed potential for reducing psychological risk.
8	Loneliness; trauma	Role of maladaptive personality traits and trauma-related symptomatology in adolescent loneliness	Empirical study with mediation analyses	Trauma-related symptoms and maladaptive personality traits played a significant role in perceived loneliness, both directly and indirectly.
9	Self-efficacy; prevention	Scouting as a strategy supporting mental health development through self-efficacy	Empirical, applied research	Engagement in scouting activities supported mental health by strengthening self-efficacy and social competence.
10	Temperament; self-injury	Relationships among temperament, emotional tension, maladaptive perfectionism, and self-injurious behavior	Empirical mediation study	Maladaptive perfectionism mediated the association between temperament and emotional tension, increasing vulnerability to self-injurious behavior.
11	Mental health screening; intervention	Development of a community-based therapeutic assessment and screening protocol for youth	Applied methodological study	A multi-level screening and assessment protocol was effective in identifying youth mental health risks and supporting early intervention.

## 3. Conclusions

This Special Issue aimed to explore and clarify the complex factors and mechanisms that were responsible for both mental health and the development of mental illness during adolescence. Specifically, it sought to identify and characterize the relationships between adolescents’ personal resources, stress-coping strategies, cultural determinants, dynamic developmental changes, and individual beliefs and cognitive schemas. The role of these factors in shaping psychological well-being, resilience, and vulnerability to mental health problems was also examined. Adolescence was selected as the target research group, due to its exceptional developmental significance and heightened psychological sensitivity [4,8]. Compared with other stages of human development, adolescence is a psychologically distinctive period that is characterized by intense biological, cognitive, emotional, and social changes, as well as the formation of identity and autonomy. At the same time, it constitutes a relatively coherent developmental category with respect to key developmental factors, thereby enabling a systematic and comparative investigation of mechanisms that influence the mental health of young people.

The studies included in this Special Issue, encompassing a wide range of psychological, social, and cultural factors, provided numerous valuable and insightful findings regarding the mental health of young people. The conclusions drawn revealed a number of important relationships that deepen the understanding of the determinants, course, and consequences of mental disorders in this population. This issue remains highly relevant, as in the context of rapid societal changes and worsening mental health indicators in the population, there is still a noticeable lack of detailed research findings concerning specific mental health markers within the adolescent group: a gap that has also been highlighted in previous research [12,17]. In this context, our findings, derived from a systematic examination of the interplay among personal, social, and cultural factors, are critical for advancing both a theoretical understanding of and practical approaches to adolescent mental health.

The main conclusions drawn from the findings of the above articles include the dynamic and significant role of emotional factors in shaping indicators of mental health in young people; the presence of close interactions between psychological factors, environmental conditions, and technological changes (e.g., social media use, technological innovations, or the Internet); and the mediating influence of various factors in the relationships between personality and mental health. Furthermore, these studies underscore the necessity of considering a range of cultural and social variables to fully understand trajectories of mental health development in adolescence. They are consistent with prior research, which has recognized that psychological factors are inextricably linked with social and cultural changes, such as strengthened family relationships, heightened awareness of social media use, and exposure to positive role models and norms [7,15,18]. To counteract negative mental health outcomes, effective coping strategies, adaptive cognitive reframing, and targeted psychoeducation may play a critical role.

The findings presented in this Special Issue, while primarily based on empirical data and their scientific interpretation, also provide a rich foundation for formulating practical recommendations that may be useful for adolescents, their families, and teams of psychiatrists, psychologists, and educators involved in youth support. Coping with mental disorders and fostering adaptation can be enhanced through targeted therapeutic interventions that address not only psychological factors but also the specific characteristics of mental disorders and the influence of environmental and cultural contexts. The results of the studies included in this Special Issue clearly demonstrate that adolescence is a unique developmental period in human life, during which broadly understood psychosocial and cultural factors dynamically shape mental health. They therefore have practical value, as they indicate potential approaches for psychological support and preventive programs for young people. This can promote coping processes and improve adolescents’ quality of life.

## References

[1] Crockett M.A., Núñez D., Martínez P., Borghero F., Campos S., Langer Á.I., Martínez V. (2025). Interventions to Reduce Mental Health Stigma in Young People: A Systematic Review and Meta-Analysis. JAMA Netw. Open.

[2] Sacco R., Camilleri N., Eberhardt J., Umla-Runge K., Newbury-Birch D. (2024). A Systematic Review and Meta-Analysis on the Prevalence of Mental Disorders among Children and Adolescents in Europe. Eur. Child Adolesc. Psychiatry.

[3] McGorry P.D., Mei C., Dalal N., Alvarez-Jimenez M., Blakemore S.J., Browne V., Killackey E. (2024). The Lancet Psychiatry Commission on Youth Mental Health. Lancet Psychiatry.

[4] Bjorklund D.F., Blasi C.H. (2026). Child and Adolescent Development: An Integrated Approach.

[5] Shorey S., Ng E.D., Wong C.H. (2022). Global Prevalence of Depression and Elevated Depressive Symptoms among Adolescents: A Systematic Review and Meta-Analysis. Br. J. Clin. Psychol..

[6] Alho J., Gutvilig M., Niemi R., Komulainen K., Böckerman P., Webb R.T., Hakulinen C. (2024). Transmission of Mental Disorders in Adolescent Peer Networks. JAMA Psychiatry.

[7] Hards E., Loades M.E., Higson-Sweeney N., Shafran R., Serafimova T., Brigden A., Borwick C. (2022). Loneliness and Mental Health in Children and Adolescents with Pre-Existing Mental Health Problems: A Rapid Systematic Review. Br. J. Clin. Psychol..

[8] Hossain M.M., Nesa F., Das J., Aggad R., Tasnim S., Bairwa M., Ramirez G. (2022). Global Burden of Mental Health Problems among Children and Adolescents during the COVID-19 Pandemic: An Umbrella Review. Psychiatry Res..

[9] Deng J., Zhou F., Hou W., Heybati K., Lohit S., Abbas U., Heybati S. (2023). Prevalence of Mental Health Symptoms in Children and Adolescents during the COVID-19 Pandemic: A Meta-Analysis. Ann. N. Y. Acad. Sci..

[10] Lin J., Guo W. (2024). Research on Risk Factors for Adolescents’ Mental Health. Behav. Sci..

[11] Daulay W. (2025). Biological Determinants of Adolescent Mental Health. J. Dow Univ. Health Sci..

[12] Kim K.M., Kim D., Chung U.S. (2020). Investigation of the Trend in Adolescent Mental Health and Its Related Social Factors: A Multi-Year Cross-Sectional Study over 13 Years. Int. J. Environ. Res. Public Health.

[13] Basu S., Banerjee B. (2020). Impact of Environmental Factors on Mental Health of Children and Adolescents: A Systematic Review. Child. Youth Serv. Rev..

[14] Carbone P. (2024). Genetic, Neurobiological, and Social Determinants of Adolescent Behavior: Risk Pathways and Protective Mechanisms. Adolescents.

[15] Orben A., Tomova L., Blakemore S.J. (2020). The Effects of Social Deprivation on Adolescent Development and Mental Health. Lancet Child Adolesc. Health.

[16] Krok D., Półtorak M. (2025). Social Media Mindsets and Well-Being in Emerging Adults: A Serial Mediation of Facebook Addiction and Stress. Brain Sci..

[17] Birrell L., Werner-Seidler A., Davidson L., Andrews J.L., Slade T. (2025). Social Connection as a Key Target for Youth Mental Health. Ment. Health Prev..

[18] Sabah A., Alduais A. (2025). Intersections of Family Expressiveness and Adolescent Mental Health: Exploring Parent–Adolescent Relationships as a Mediator. Ment. Health Soc. Incl..

